# Depressive, anxiety, and burnout symptoms on health care personnel at a month after COVID-19 outbreak in Indonesia

**DOI:** 10.1186/s12889-021-10299-6

**Published:** 2021-01-28

**Authors:** Deni Kurniadi Sunjaya, Dewi Marhaeni Diah Herawati, Adiatma Y. M. Siregar

**Affiliations:** 1 Department of Public Health, Faculty of Medicine, Universitas Padjadjaran, Jalan Eyckman No 38, Bandung, West Java 40161 Indonesia; 2 Department of Economic, Faculty of Economic and Business, Universitas Padjadjaran, Bandung, West Java 40161 Indonesia

**Keywords:** Depressive, Anxiety, Burnout symptoms, Health care personnel, COVID-19, Indonesia

## Abstract

**Background:**

Health care personnel (HCP) who demonstrated close contact with Corona virus disease (COVID-19) patients might experience a higher risk of infection and psychological problems. This study aims to explore depressive, anxiety, and burnout symptoms among HCP with a higher risk for psychological trauma.

**Methods:**

This study was a cross-sectional study using secondary data from an online assessment, which was conducted 1 month after the COVID-19 outbreak. A total of 544 respondents from 21 provinces in Indonesia were included. Data on depressive, anxiety, and burnout symptoms were transformed first using the Rasch model and then categorized. Data from HCP in the higher risk group and the lower risk group were analyzed.

**Results:**

A higher percentage of HCP experiencing depressive symptoms (22.8%), anxiety (28.1%), and burnout (26.8%) are found in the higher risk group. The chance for the higher risk group’s HCP to present with moderate and severe depressive symptoms, anxiety, and burnout are: 5.28 (Confidence interval (CI): 2.01–13.89; *p* < 0.05), 1.36 (CI: 0.09–1.96; *p* >  0.05), and 3.92 (CI: 2.08–7.40; *p* < 0.05) times higher, respectively. The probability for patient-induced burnout is 2.13 (CI: 1.51–3.007; *p* < 0.05) times higher and highest among the other burn out dimensions. The depressive symptoms complained were similar between groups: loneliness, sleep disturbances, difficulty concentrating, and inability to initiate activities. Loneliness demonstrates the highest logit value among the symptoms.

**Conclusions:**

HCP with direct contact and responsibility to treat COVID-19 patients exhibit a higher risk to experience depressive symptoms and burnout. Communication with peers and staying in contact with family needs to be encouraged. Psychological well-being should be considered for high-risk HCP. Incentive or insurance guaranteed by the government or institution is essential as a reward and compensation during this period.

## Background

The Indonesian government publicly announced its two first COVID-19 cases on March 2, 2020. Ten days later, the World Health Organization (WHO) increased the COVID-19 status into a pandemic [1, 2], as this disease has been spreading and infecting most of the countries worldwide. In Indonesia, the total infected cases reported on May 6, 2020 was 12,438 cases with 895 deaths [3]. Sadly, as of September 2020, 184 HCP died due to the pandemic [4].

HCP who demonstrated close contact with COVID-19 patients, might experience a higher risk of infection and psychological problems. Studies show that work demands and lack of social supports increase the risk of depression and job burnout [5–7]. Medical doctors and nurses are at high-risk for emotional exhaustion and infection due to disease exposure, psychological distress [8], and shortage in personal protective equipment [9–12]. Also, nurses who are treating critically ill patients exhibit a considerable risk of secondary traumatic stress [13–16].

The risk for anxiety, depression, and stress is significant for health professionals [17–19]. A study in Wuhan indicated that health professionals treating patients with COVID-19 are at a considerable, if not excessive, pressure due to job demands, fatigue, and frustration, accompanied by isolation and lack of contact with their family. Besides, inadequate protective equipment might lead them to contamination and infection [20].

Before the pandemic, health professionals were faced with a high risk for anxiety, depression, and fatigue due to their jobs [21, 22]. Researchers reported that doctors complain about experiencing anxiety (25.67%), depressive symptoms (28.13%), and both problems (19.01%) [23].

Styta et al. argues that the risk factors for psychologic pressure among health professionals are their perceptions on their job’s risks, working in a high-risk environment, the diseases’ effects on their working life, and the possibility for being infected by the patients [24]. Chai et al. explained that the health professionals worry about their as well as their family’s safety, particularly when treating a deceased patient [25]. Conversely, adequate and strict infection control protocol, complete safety equipment, and solemn recognition and adequate appreciation from their institutions and government on their role in managing patients with COVID-19 are essential to improve their psychological condition [25].

Anxiety is a psychological condition characterized by cognitive, somatic, emotional, and behavior components [26], various severity level [27], and association with economic and social problems [28]. A depressive disorder is a common mental health problem with mood or feeling disturbances, lack of interest or happiness, guilty feeling or low self-esteem, sleep disturbances, less appetite, low energy, and lousy concentration ability [29]. Sadness and rejection are the most salient emotional symptoms in depressive disorder [30]. Depressive signs and symptoms include depressive, guilty, and unworthy feelings, helplessness and desperation, psychomotor problems, lack of appetite, and sleep disturbances [31]. Studies show that more than 30% of healthcare workers suffer from any of such psychological condition, and it is significantly associated with the currently more prevalent physical symptoms (e.g. headache, neck stiffness) during the Covid-19 pandemic [32, 33]. In addition, burnout syndrome, characterized by three dimensions, e.g. emotional exhaustion, depersonalization and personal accomplishment and typically occurs in helping professions such as HCP [34, 35] correlates to lack of performances [36, 37], which might further include social withdrawal [38, 39]. Indeed, studies have shown that health care workers experience high burnout prevalence rate, e.g. 11.23% among nurses and 51% among residents, globally [40, 41].

This study aims to explore the depressive, anxiety, and burnout symptoms among HCP as a risk of psychological trauma in handling Covid-19. Our study fills the still relatively large gap of this currently under researched topic [42]. Our findings present the evidence on Indonesian HCP’s psychological condition during COVID-19 pandemic, an important factor to be addressed within the policies combating the pandemic.

## Methods

### Study design

This study was a cross-sectional approach using the secondary data provided by a survey conducted by the Center of Economics Development Study (CEDS), Universitas Padjadjaran, Indonesia. The CEDS’ assessment was conducted after the first month of the COVID-19 outbreak in Indonesia. This assessment covered economic, social, health, and psychosocial aspects.

The study population consisted of general practitioners, emergency doctors, and doctors in various specialists, dentists, nurses, midwives, analysts, pharmacists, and public health practitioners. The health care centers participated were widely distributed, including state-owned/public and Private Hospitals, and Primary Health Care Centers (PHCC) which are private clinic and private doctor practice, and public Community Health Centers (CHC) or *Puskesmas.*

### Data collection

The survey conducted by CEDS applied convenience (non-probability) sampling. Data collection was conducted online using google form. Informed consent was asked at the beginning of the online questionnaire that contains the explanation, aim, participants, anonymity, and volunteerism of the survey.

The questionnaire was distributed for a month (April 2020) through the associations of: profession (doctors, dentists, nurses, midwives), hospitals, clinics, and primary health centers. As many as 569 HCPs participated in this assessment. A total of 563 subjects (98.9%) were collected from several cities and districts. However, only 544 samples (96.6%) met the inclusion criteria and were analyzed.

This study’s variables included depressive symptoms, anxiety, and burnout. Depressive symptoms were assessed using the Centre for Epidemiological Studies Depression Scale (CESD R-10) scale consisting of 10 questions. Anxiety was evaluated using the Zung Anxiety Scale (ZAS) (20 questions), whereas the Burnout Inventory (BOI), which is comprised of personal, work, and patient dimensions in a total of 19 questions, was utilized for evaluating burnout. The three instruments are regularly used in Indonesia in mental health clinical practices, management of organizations, and studies [43–47].

### Data analysis

The Rasch model analysis was conducted using the Winsteps application to transform the data [48] and obtain each participant’s Log Odds Unit (logit) score. The mean logit score cut off indicated the risk of tendency to experience depressive symptoms, anxiety, and burnout. Furthermore, we used standard deviation for moderate and severe level cut off point. Burn out was explored further for each dimension. Similarly, depression related items were explored using differential item functioning (DIF) [49].

All instruments and measurement results were reported to be valid and reliable. The validity and reliability of the instruments are presented in Table 1.

**Table 1:** Instruments reliability and model fit

Psychometric Attribute	Instrument		
	CESDR-10	ZAS	BOI
Number of items	10	20	19
Outfit Mean Square			
*Mean*	0.99	1.02	1.00
*Standard Error Measurement (SEM)*	0.13	0.07	0.29
Separation	6.78	11.89	9.65
Reliability	0.98	0.99	0.99
Cronbach’s alpha	0.84	0.91	0.93
Uni-dimensionality			
*Raw variance*	58.2%	46.1%	49.6%
*Unexplained variance 1st contrast*	2.04	2.47	3.23

HCP were divided into two groups (available in the data source): those with a higher risk for trauma, which are positive for having any (direct) contact with patients with COVID-19 or in duty for treating the patients (i.e., the higher risk group), and those with a lower risk for trauma (i.e., the lower risk group). The depressive, anxiety, and burnout symptoms score were compared and analyzed between both groups. Odds Ratio (OR) were calculated using crosstab analysis, and adjusted OR were estimated using binary logistic regression.

## Results

In the group of nurses, a higher percentage of nurses (62.1%) are found to exhibit a risk for psychological trauma in comparison to other professions as shown in Table 2. HCP working in both public and private centers demonstrate a higher chance of trauma when being compared to those who are working in only one institution.

**Table 2:** Demographic characteristics of study participants

	All Participants		Higher Risk		
	N	%	N	% (between group)	% (pop.)
Gender					
Male	124	22.8	72	58.1	13.2
Female	420	77.2	219	52.1	40.3
Age					
< 30	165	30.3	80	48.5	14.7
30–39	182	33.5	97	53.3	17.8
40–50	140	25.7	84	60.0	15.4
> 50	57	10.5	30	52.6	5.5
Profession					
Doctor	144	26.5	76	52.8	14.0
Nurse	124	22.8	77	62.1	14.2
Others	276	50.7	138	50.0	25.4
Working’s place					
Hospital	173	31.8	94	54.3	17.3
PHCC	52	9.6	26	50.0	4.8
CHC (*Puskesmas*)	258	47.4	146	56.6	26.8
Mixed (CHC + PHCC)	26	4.8	12	46.2	2.2
Others	35	6.4	13	37.1	2.4
Public owned	387	71.1	215	55.6	39.5
Private owned	115	21.1	51	44.3	9.4
Both Public & Private	42	7.7	25	59.5	4.6
Marriage status					
Not married	118	21.7	61	51.7	11.2
Married	426	78.3	230	54.0	42.3

Table 3 shows that HCP with a history of contact with COVID-19 patients exhibit a higher risk for (psychological) trauma compared to the other group (depression: 22.8% vs 13.4%, anxiety: 28.1% vs 21.5%, and burnout: 26.6% vs 15.8%). The OR for depression, anxiety, and burnout (all level) are 1.8, 1.3, and 1.48 times, respectively. Moderate and severe psychologic disturbances are higher in the higher risk group (depression: 5.15% vs 0.92%, anxiety: 10.5% vs 7.5%, and burnout: 9.4% vs 2.4%). The OR for moderate and severe depression, anxiety, and burnout are 5.3 (CI 95%: 2.007–13.892), 1.26 (0.089–1.960), and 3.92 (2.080–7.401), respectively. Working place is a confounding factor that has a significant correlation. Adjusted OR shows a small difference on depressive symptoms, anxiety and burn out at all or moderate-severe level.

**Table 3:** Depressive, anxiety, and burnout symptoms in HCP in higher and lower risk group

	Depressive Symptom (%)		Anxiety (%)		Burnout (%)	
	All Level	Moderate-Severe	All Level	Moderate-Severe	All Level	Moderate-Severe
All HP	36.2	6.1	49.6	18.0	42.4	11.8
Higher risk	22.8	5.15	28.1	10.5	26.6	9.4
Lower risk	13.4	0.92	21.5	7.5	15.8	2.4
OR	1.83	5.28	1.29	1.26	1.93	3.92
CI (95%)	1.28–2.61	2.01–13.89	0.92–1.36	0.09–1.96	1.36–2.73	2.08–7.40
*p* value	0.001	0.001	> 0.05	> 0.05	0.000	0.000
OR (adj.)	1.81	5.82	1.29	1.28	1.90	3.78
CI (95%)	1.26–2.61	2.18–15.56	0.92–1.83	0.82–1.99	1.34–2.70	1.99–7.16
*p* value	0.001	0.000	> 0.05	> 0.05	0.000	0.000

Table 4 shows personal, work, and patient dimensions in the burnout. It indicates that the risk group is more prone to trauma. The OR for personal dimension is 1.43, whereas the work and patient dimensions are 1.34 and 2.13, respectively; the burnout score for patient dimension is higher compared to the rest.

**Table 4:** Burnout for personal, work, and patient dimensions

Health Personal (%)	Personal (%)	Work (%)	Patient (%)
All Subjects	52.75	52.75	46.71
Risk Group	28.77	29.84	29.31
Non-risk Group	23.98	22.91	17.41
OR	1.43	1.34	2.13 (1.51–3.007; *p* < 0.05)

**Fig. 1:**
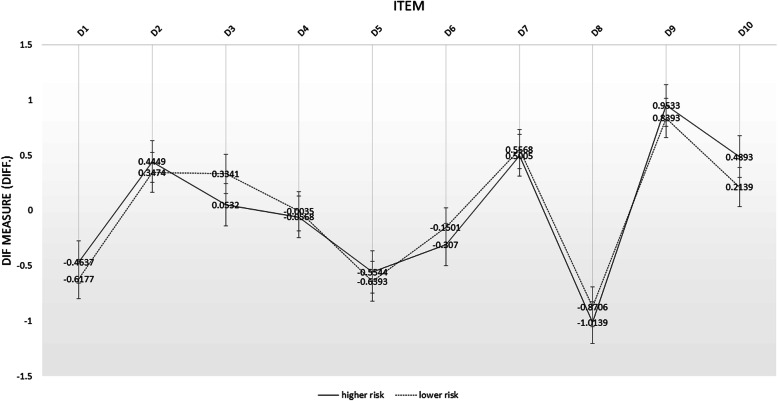
Person DIF plot of depressive symptoms of higher and lower risk of health care professionals. This figure shows the DIF plot of depressive symptoms among health care professionals for each CESDR-10 item, differentiated by risk level

Figure 1 shows depressive symptoms experienced by HCP in both groups are somewhat similar. There is no bias caused by risk level on all items. Both groups reported that loneliness (D9), sleep disturbances (D7), inability to initiate activities (D2), and difficulty concentrating (D10) as items that have high logit values. Among all questions, depressive feeling (D3) is prominent in the lower risk group’s members. Loneliness (D9) is the highest logit value among the symptoms and is slightly higher in the higher risk group.

## Discussion

Results of this study indicate that nurses and other HCP working in two institutions demonstrate a higher risk of trauma. A similar indication was found in Coetzee and Klopper’s, Mealer and Jones’, as well as Austin et al.’s studies [50–52]. A review published by Sporrthy showed that nurses exhibit higher anxiety and depression symptoms in comparison to doctors [53]. Nonetheless, a study in Singapore found that unmarried (single) doctors are at a higher risk for psychological problems than married nurses [54].

This study shows that HCPs in the higher risk group are more prone to depression, anxiety, and burnout in comparison to the lower risk group’s members. Depression exhibits a higher chance to raise both the risks of anxiety and burnout. Our result is in line to Nushad et al.’s study, which showed that HCPs in an emergency department and nurses in intensive rooms and infectious diseases ward exhibit a higher risk for adverse psychiatric effects [55]. Another study argued that 23.7% intensive care unit doctors in France experience depression [56]. Similarly, Bai et al. indicated that 5% of health professionals experienced acute stress disorders, 20% experienced stigmatization and refusal from their environment, and 9% of them resign from jobs [57].

This study shows that 6.1% of HCPs present with moderate-severe depressive symptoms. This figure is lower than the previous study on Indonesian adults by Peltzer and Pengpid. They found that 15 and 6.9% Indonesian adults experience moderate and severe depressive symptoms, respectively, and 21.8% experience moderate to severe depressive symptoms [58]. This difference might be because the HCPs are used to treating patients. However, Al-Maddah et al. argued that moderate to severe depressive symptoms prevalence in doctors are approximately 20% [59]. The variation in those studies’ results as compared to our study is probably owing to the differences in instruments utilized; whereas the Al-Maddah et al. used Beck Depression Inventory-2 (BDI-2), and this study utilized CESDR-10.

The percentage of HCPs experiencing moderate-severe anxiety in this study was higher (18%) than depressive symptoms (6.1%) and burnout (11.8%). A lower percentage was reported from Australian health care professionals by Kilkkinen. Although anxiety is more common to other problems (similar to this current study), a lower percentage of each mental problem (3.7 and 3% for anxiety and depressive symptoms, respectively) was found in their study [60]; suggesting that the COVID-19 pandemic might have caused higher anxiety and depressive symptoms prevalence among HCP compared to the general population.

The percentage of HCPs experiencing burnout in this study is considerably high, which is similar to the findings of Maslach et al. who compared health professionals to general workers [61]. A study in the Netherlands showed that burnout in the doctors population is higher than what is found in general population [62, 63]. The current study’s results also demonstrating that burnout due to patients are higher than what captured in personal and work dimensions, indicating that patients with COVID-19 might become a trigger for burnout in health professionals. In line with our study, Holmqvist and Jeanneau explained that patient dimension, such as negative feeling on patients, demonstrates a higher correlation with the doctor’s burnout [64]. This argument was supported by Bressi et al., who argued that working with patients and their families are associated with burnout, notably when higher, unrealistic hope for the treatment result is involved [65].

Negative psychological impact of the pandemic occurred for doctors who work at high risk workplaces [66, 67]. In these studies, HCPs are having potential risk of suffering from negative psychological impact if there is a disaster such as pandemic. The impact became larger if the workplace has higher risk.

Our result indicates that depressive feeling exhibits a higher incidence in the risk group. Similarly, Fried, in his study, argued that depressive feelings and loss of interest are both high contributing factors [68]. Loneliness is the stand out symptom for health personnel. This feeling of solitude needs to be understood because it is often unsaid and neglected even by themselves. An irony of loneliness in a crowd of COVID-19 issues. They need to talk and communicate whenever they want it. Spouse, family, friends need to maintain contact and provide time to deal with these feelings before the burden of the feeling increases. Screening and monitoring needs to be done routinely, and responses are given to health workers who are overloaded with psychiatric disorders. Ho et al suggest to conduct cognitive behavior therapy and mindfulness-based therapy to cope with the psychological impact of the pandemic [69].

HCPs treating and demonstrating contact with patients with COVID-19 in this study experience significantly higher depressive symptoms and burnout than the other group. The percentage of HCPs experiencing anxiety was also higher despite insignificant differences in the chance for it to raise. Anxiety may increase as a consequence of pandemic condition, not only due to the management of the epidemic origin. Therefore, HCPs who are in contact with patients with COVID-19 exhibit a higher risk of psychologic trauma from their works.

The institution should consider providing appropriate incentive and compensation for this risk. Epidemic or pandemic condition is a public health problem with enormous consequences. Therefore, incentives and compensation are obligatory for the government. Sacrifices exhibited by HCPs working in a high-risk situation should be given rewards from the public or the government, without differentiating between those working in public or private institutions.

### Limitation

This study demonstrates several limitations. The secondary data used were limited to HCPs working in a pandemic situation. Thus, these data may not represent the whole condition of Indonesian health care providers. Our cross-sectional design does not compare our results with the state of individuals’ mental health before pandemic, thus inferring causality should be made cautiously. Online assessment may not attain respondents with very severe depression as they will not be able to communicate well.

## Conclusions

The COVID-19 pandemic raised psychological disturbances among the people, including HCPs. HCPs who are exhibiting contact and treating Covid-19 patients demonstrate a higher psychologic risk, in terms of depression and burnout, which varies from mild to severe condition compared to those who do not. Loneliness is the most prominent depressive symptom in HCPs. Communication with peers and staying in contact with family needs to be encouraged. Psychological well-being should be considered for higher risk HCPs during this pandemic. Therefore, policies being developed to combat Covid-19 should robustly acknowledge this aspect as it is currently receiving lack of attention. Although incentives and insurance from the government or health institution are essential as a reward and compensation for HCPs, providing preventive interventions in regards to mental illness within all type of health care facilities should be considered a priority to ensure the sustainability of the services provided by HCPs.

## Data Availability

All calculation data generated and/or analyzed during the current study are available from the corresponding author on reasonable request.

## Abbreviations

BDI-2: Beck Depression Inventory-2
BOI: Burnout Inventory
CEDS: Center of Ecomonics Development Study Universitas Padjadjaran
CESDR-10: Centre for Epidemiological Studies Depression Scale
CHC: Community Health Centers
CI: Confidence Interval
COVID-19: Corona Virus Disease
DIF: Differential Item Functioning
HCP: Health Care Personnel
Logit: Log Odds Unit
OR: Odds Ratio
PHCC: Primary Health Care Centers
SEM: Standard Error Measurement
WHO: World Health Organization
ZAS: Zung Anxiety Scale

## References

[1] World Health Organization. Coronavirus disease (covid-19) outbreak - WHO announces COVID-19 outbreak a pandemic. 2020. http://www.euro.who.int/en/ health-topics/health-emergencies/coronavirus-covid-19/news/news/2020/3/who-announces-covid-19 outbreak-a-pandemic.

[2] Day M (2020). Covid-19: surge in cases in Italy and South Korea makes pandemic look more likely. BMJ.

[3] Yurianto A (2020). Media center Gugus Tugas Penanganan Percepatan Covid-19 Badan Nasional Penanggulangan Bencana (BNPB).

[4] CNN Indonesia (2020). IDI: 184 health care professional died due to corona pandemic.

[5] Bonde JPE (2008). Psychosocial factors at work and risk of depression: a systematic review of the epidemiological evidence. Occup Environ Med.

[6] Sinokki M, Hinkka K, Ahola K, Koskinen S, Kivimäki M, Honkonen T (2009). The association of social support at work and in private life with mental health and antidepressant use: the health 2000 study. J Affect Disord.

[7] Chirico F (2016). Job stress models for predicting burnout syndrome: a review. Ann Ist Super Sanita.

[8] Chirico F, Nucera G, Magnavita N (2020). Protecting the mental health of healthcare workers during the COVID-19 emergency. BJPsych Int.

[9] Guangming Online (2020). Beijing: central steering group: over 3,000 medical staff in Hubei were infected in the early stage of the epidemic, currently no infection reports among medical aid staff.

[10] World Health Organization. Shortage of personal protective equipment endangering health workers worldwide. Geneva: World Health Organization; 2020. [cited 2020 March 3].

[11] Magnavita N, Sacco A, Nucera G, Chirico F (2020). First aid during the COVID-19 pandemic. Occup Med.

[12] Magnavita N, Chirico F (2020). Headaches, personal protective equipment, and psychosocial factors associated with COVID-19 pandemic. Headache J Head Face Pain.

[13] Mealer ML, Shelton A, Berg B, Rothbaum B, Moss M (2007). Increased prevalence of post-traumatic stress disorder symptoms in critical care nurses. Am J Respir Crit Care Med.

[14] Molloy J, Evans M, Coughlin K (2015). Moral distress in the resuscitation of extremely premature infants. Nurs Ethics.

[15] Cook KA, Mott S, Lawrence P, Jablonski J, Grady MR, Norton D (2012). Coping while caring for the dying child: nurses’ experiences in an acute care setting. J Pediatr Nurs.

[16] Quinal L, Harford S, Rutledge DN (2009). Secondary traumatic stress in oncology staff. Cancer Nurs.

[17] Wu KK, Chan SK, Ma TM (2005). Posttraumatic stress, anxiety, and depression in survivors of severe acute respiratory syndrome (SARS). J Trauma Stress.

[18] Lai J, Ma S, Wang Y, Cai Z, Hu J, Wei N (2020). Factors associated with mental health outcomes among health care workers exposed to coronavirus disease 2019. JAMA Netw Open.

[19] Zhang WR, Wang K, Yin L, Zhao WF, Xue Q, Peng M (2020). Mental health and psychosocial problems of medical health workers during the COVID-19 epidemic in China. Psychother Psychosom.

[20] Kang L, Li Y, Hu S, Chen M, Yang C, Yang BX (2020). The mental health of medical workers in Wuhan, China dealing with the 2019 novel coronavirus. Lancet Psychiatry.

[21] Wallace JE (2012). Mental health and stigma in the medical profession. Health.

[22] Arnetz BB (2001). Psychosocial challenges facing physicians of today. Soc Sci Med.

[23] Gong Y, Han T, Chen W, Dib HH, Yang G, Zhuang R (2014). Prevalence of anxiety and depressive symptoms and related risk factors among physicians in China: a cross-sectional study. PLoS One.

[24] Styra R, Hawryluck L, Robinson S, Kasapinovic S, Fones C, Gold WL (2008). Impact on health care workers employed in high-risk areas during the Toronto SARS outbreak. J Psychosom Res.

[25] Cai H, Tu B, Ma J, Chen L, Fu L, Jiang Y (2020). Psychological impact and coping strategies of frontline medical staff in Hunan between January and march 2020 during the outbreak of coronavirus disease 2019 (COVID) in Hubei, China. Med Sci Monit.

[26] Ahmed I, Banu H, Al-Fageer R, Al-Suwaidi R (2009). Cognitive emotions: depression and anxiety in medical students and staff. J Crit Care.

[27] Baxter AJ, Scott KM, Vos T, Whiteford HA (2013). Global prevalence of anxiety disorders: a systematic review and meta-regression. Psychol Med.

[28] Kessler RC, Greenberg PE (2000). The economic burden of anxiety and stress disorders. Neuropsychopharmacology.

[29] World Health Organization. Depression. Geneva: WHO; 2012. https://www.who.int/news-room/fact-sheets/detail/depression.

[30] Nolen-Hoeksema S, Fredrickson BL, Loftus GR, Wagenaar WA. Atkinson & Hilgard’s introduction to psychology. 15th ed. UK: Wadsworth Pub Co; 2009.

[31] Zhang W, O’Brien N, Forrest JI, Salters KA, Patterson TL, Montaner JSG (2012). Validating a shortened depression scale (10 item CES-D) among HIV-positive people in British Columbia, Canada. PLoS One.

[32] Chew NWS, Lee GKH, Tan BYQ, Jing M, Goh Y, Ngiam NJH (2020). A multinational, multicentre study on the psychological outcomes and associated physical symptoms amongst healthcare workers during COVID-19 outbreak. Brain Behav Immun.

[33] Tan BYQ, Chew NWS, Lee GKH, Jing M, Goh Y, Yeo LLL (2020). Psychological impact of the COVID-19 pandemic on health care workers in Singapore. Ann Intern Med.

[34] Chirico F, Police S (2017). Burnout syndrome and depression are not the same thing. Br J Psychiatry.

[35] Chirico F (2017). Is burnout a syndrome or an occupational disease? Instructions for occupational physicians. Epidemiol Prev.

[36] Ruotsalainen JH, Verbeek JH, Mariné A, Serra C. Preventing occupational stress in healthcare workers. Cochrane Database Syst Rev. 2014;12:CD002892. 10.1002/14651858.CD002892.pub3 25391582

[37] Maslach C, Schaufeli WB, Leiter MP (2001). Job burnout. Annu Rev Psychol..

[38] Alarcon GM (2011). A meta-analysis of burnout with job demands, resources, and attitudes. J Vocat Behav.

[39] Kim H, Kao D (2014). A meta-analysis of turnover intention predictors among U.S. child welfare workers. Child Youth Serv Rev.

[40] Low ZX, Yeo KA, Sharma VK, Leung GK, McIntyre RS, Guerrero A (2019). Prevalence of burnout in medical and surgical residents: a meta-analysis. Int J Environ Res Public Health.

[41] Woo T, Ho R, Tang A, Tam W (2020). Global prevalence of burnout symptoms among nurses: a systematic review and meta-analysis. J Psychiatr Res.

[42] Tran BX, Ha GH, Nguyen LH, Vu GT, Hoang MT, Le HT (2020). Studies of novel coronavirus disease 19 (COVID-19) pandemic: a global analysis of literature. Int J Environ Res Public Health.

[43] Nursalam N, Fibriansari RD, Yuwono SR, Hadi M, Efendi F, Bushy A (2018). Development of an empowerment model for burnout syndrome and quality of nursing work life in Indonesia. Int J Nurs Sci.

[44] Wulandari P, Hidayat R (2020). General anxiety disorder-related coronavirus disease-19 outbreak in Indonesia: a case report. Maced J Med Sci.

[45] Marthoenis M, Meutia I, Fathiariani L, Sofyan H (2018). Prevalence of depression and anxiety among college students living in a disaster-prone region. Alexandria J Med.

[46] Fahmi M, Panjaitan NA, Habibie I, Siregar AYM, Amarullah G (2019). Rahma, et al. does your neighborhood protect you from being depressed? A study on social trust and depression in Indonesia. BMC Public Health.

[47] Yestiana Y, Kurniati T, Hidayat AAA (2019). Predictors of burnout in nurses working in inpatient rooms at a public hospital in Indonesia. Pan Afr Med J.

[48] Boone WJ, Staver JRYM (2014). Rasch analysis in the human sciences.

[49] Sumintono B, Widhiarso W (2014). Application of Rasch model for social science research.

[50] Coetzee SK, Klopper HC (2010). Compassion fatigue within nursing practice: a concept analysis. Nurs Health Sci.

[51] Mealer M, Jones J (2013). Posttraumatic stress disorder in the nursing population: a concept analysis. Nurs Forum.

[52] Austin W (2012). Moral distress and the contemporary plight of health professionals. HEC Forum.

[53] Spoorthy MS (2020). Mental health problems faced by healthcare workers due to the COVID-19 pandemic–a review. Asian J Psychiatr.

[54] Chan AOM, Chan YH (2004). Psychological impact of the 2003 severe acute respiratory syndrome outbreak on health care workers in a medium size regional general hospital in Singapore. Occup Med.

[55] Naushad VA, Bierens JJLM, Nishan KP, Firjeeth CP, Mohammad OH, Maliyakkal AM (2019). A systematic review of the impact of disaster on the mental health of medical responders. Prehosp Disaster Med.

[56] Embriaco N, Hraiech S, Azoulay E, Baumstarck-Barrau K, Forel JM, Kentish-Barnes N (2012). Symptoms of depression in ICU physicians. Ann Intensive Care.

[57] Bai YM, Lin CC, Lin CY, Chen JY, Chue CM, Chou P (2004). Survey of stress reactions among health care workers involved with the SARS outbreak. Psychiatr Serv.

[58] Peltzer K, Pengpid S (2018). High prevalence of depressive symptoms in a national sample of adults in Indonesia: childhood adversity, sociodemographic factors and health risk behaviour. Asian J Psychiatr.

[59] Al-Maddah EM, Al-Dabal BK, Khalil MS (2015). Prevalence of sleep deprivation and relation with depressive symptoms among medical residents in king Fahd University hospital, Saudi Arabia. Sultan Qaboos Univ Med J.

[60] Kilkkinen A, Kao-philpot A, O’neil A, Philpot B, Reddy P, Bunker S (2007). Prevalence of psychological distress, anxiety and depression in rural communities in Australia. Aust J Rural Health.

[61] Maslach C, Leiter MP (2016). Understanding the burnout experience: recent research and its implications for psychiatry. World Psychiatry.

[62] Twellaar M, Winants Y, Houkes I (2008). How healthy are Dutch general practitioners? Self-reported (mental) health among Dutch general practitioners. Eur J Gen Pract.

[63] Shanafelt TD, Boone S, Tan L, Dyrbye LN, Sotile W, Satele D (2012). Burnout and satisfaction with work-life balance among US physicians relative to the general US population. Arch Intern Med.

[64] Holmqvist R, Jeanneau M (2006). Burnout and psychiatric staff’s feelings towards patients. Psychiatry Res.

[65] Bressi C, Porcellana M, Gambini O, Madia L, Muffatti R, Peirone A (2009). Burnout among psychiatrists in Milan: a multicenter survey. Psychiatr Serv.

[66] Al Sulais E, Mosli M, AlAmeel T (2020). The psychological impact of COVID-19 pandemic on physicians in Saudi Arabia: a cross-sectional study. Saudi J Gastroenterol.

[67] Matsuishi K, Kawazoe A, Imai H, Ito A, Mouri K, Kitamura N (2012). Psychological impact of the pandemic (H1N1) 2009 on general hospital workers in Kobe. Psychiatry Clin Neurosci.

[68] Fried EI, Nesse RM (2014). The impact of individual depressive symptoms on impairment of psychosocial functioning. PLoS One.

[69] Ho CS, Chee CY, Ho RC (2020). Mental health strategies to combat the psychological impact of COVID-19 beyond paranoia and panic. Ann Acad Med Singap.

